# Factors Associated with Increased Analgesic Use in German Women with Endometriosis during the COVID-19 Pandemic

**DOI:** 10.3390/jcm11195520

**Published:** 2022-09-21

**Authors:** Roxana Schwab, Kathrin Stewen, Tanja Kottmann, Mona W. Schmidt, Katharina Anic, Susanne Theis, Bashar Haj Hamoud, Tania Elger, Walburgis Brenner, Annette Hasenburg

**Affiliations:** 1 Department of Obstetrics and Gynecology, University Medical Center of the Johannes Gutenberg University Mainz, Langenbeckstr. 1, 55131 Mainz, Germany; 2 CRO Dr. med Kottmann GmbH & Co. KG, 59077 Hamm, Germany; 3 Department for Gynecology, Obstetrics and Reproductive Medicine, Saarland University Hospital, 66421 Homburg, Germany

**Keywords:** chronic pelvic pain, endometriosis, analgesic use, multimodal treatment, COVID-19 pandemic

## Abstract

(1) Background: Endometriosis is a frequent chronic pain condition in women of fertile age. Pain management with analgesics is frequently used by women with endometriosis. During the COVID-19 pandemic, access to health services was temporarily restricted in various countries for persons without serious conditions, resulting in increased physical and mental health issues. The present study was conducted in order to assess the risk factors predicting increased analgesic intake by women with endometriosis during the COVID-19 pandemic. (2) Methods: The increased intake of over-the-counter (OTC) and prescription-only (PO) analgesics was assessed with an anonymous online questionnaire, along with demographic, pandemic-specific, disease-specific, and mental health characteristics. Anxiety and depression were assessed with the Generalized Anxiety Disorder Scale (GAD-2) and the Patient Health Questionnaire for Depression (PHQ-2), respectively. Pain-induced disability was assessed with the pain-induced disability index (PDI). (3) Results: A high educational level (OR 2.719; 95% CI 1.137–6.501; *p* = 0.025) and being at higher risk for depressive disorders, as measured by PHQ-2 ≥ 3 (OR 2.398; 95% CI 1.055–5.450; *p* = 0.037), were independent risk factors for an increased intake of OTC analgesics. Current global pain-induced disability (OR 1.030; 95% CI 1.007–1.054; *p* = 0.010) was identified as a risk factor for an increased intake of PO pain medication. The degree of reduction in social support and in social networks were independent predictors of an increased intake of PO analgesics in a univariate logistic regression analysis, but lost significance when adjusted for additional possible influencing factors. (4) Conclusions: In this population, an increased intake of OTC analgesics was related to a higher educational level and having a depressive disorder, while a higher pain-induced disability was an independent risk factor for an increased intake of PO analgesics. Pandemic-specific factors did not significantly and independently influence an increased intake of analgesics in women with endometriosis during the first wave of the COVID-19 pandemic in Germany. Healthcare providers should be aware of the possible factors related to increased analgesic use in women with endometriosis in order to identify persons at risk for the misuse of pain medication and to prevent potential adverse effects.

## 1. Introduction

Endometriosis is a common medical condition caused by the presence of endometrial-like tissue outside the uterine cavity, affecting up to 10–15% of women of fertile age [1]. Women with endometriosis experience a variety of different pain symptoms, such as dysmenorrhea, dyspareunia and chronic pain [1]. After initial diagnosis via laparoscopy and excision of the endometriotic lesions, pain persistence or recurrence is experienced by many patients [2,3]. The multidimensional chronic pain experience may lead to pain-induced disability in almost all areas of daily life [4]. As a result of the long diagnostic delay [5], women with endometriosis frequently use analgesics in order to mitigate pain and pain-induced disability long before the actual diagnosis, as well as after initial or recurrent surgical or hormonal therapy [6,7,8]. Current data show that up to 75% of women with endometriosis have admitted to the use of over-the-counter (OTC) pain medication, while only up to 36.9% reported the use of prescription-only (PO) analgesics [9,10].

Chronic pain is a major healthcare concern [11]. Chronic pain is defined by the International Association for the Study of Pain (IASP) as an unpleasant sensation that persists for longer than three months [12]. Therapy with analgesics is one of the main pillars of the treatment of chronic pain. A European survey among patients with chronic pain revealed that up to 47% had taken non-prescription medication in the last six months, and 52% were taking prescription medicines for their pain [11]. Self-care management and self-medication is common in patients with chronic pain [11,13]. The WHO describes self-medication as the selection and use of either OTC or PO analgesics in order to treat self-recognized illnesses or symptoms [14]. Dependent on the specific national authorization for analgesics, the medication can be sold directly to the consumer as OTC pain medication, or to consumers with a valid prescription as PO medication [13]. To date, several factors which influence the consumption of analgesics have been identified in different settings, such as demographic characteristics or mental health issues [15,16].

The SARS-CoV-2 virus was first identified at the end of 2019 in China, causing the disease COVID-19. The World Health Organization declared the outbreak of a pandemic on 11 March 2020 [17]. In order to prevent the virus from spreading, governments worldwide imposed measures. In Germany, the first widespread social distancing measures were implemented by the government at the end of March 2020 [17,18]. Access to health services was restricted for persons without serious conditions, such as cancer [19], creating new obstacles for individuals with chronic pain, such as women with endometriosis, to seek the alleviation of pain symptoms and an improvement in their quality of life [20].

In order to identify women with endometriosis at risk of increased analgesics use or misuse during stressful life events, such as the COVID-19 pandemic, we assessed the influence of various demographic, disease-specific, and pandemic-specific factors, as well as mental health, with respect to the increased use of pain medication.

## 2. Materials and Methods

### 2.1. Study Design

The anonymous online questionnaire was accessible between 6th and 27th of April 2020 for members of the Facebook internet platforms of German endometriosis patient support groups. Aside from questions related to demographics (age, marital status, living alone, and educational level) and disease-specific questions (time since endometriosis diagnosis, age at diagnosis, diagnostic delay of endometriosis, pain characteristics, pain intensity, and pain-induced disability), the questionnaire included pandemic parameters (duration of limited social networks, being in isolation or quarantine, and the level of reduction in social contacts), as well as questions regarding anxiety, depression and resilience.

The visual pain scale (VAS) was employed to assess the current pain intensity (VAS_C_), as well as the pain intensity prior to the implementation of social distancing measures (VAS_P_) [21]. Moreover, the pain was divided into three categories: “no pain to low pain” (VAS = 0–44), “moderate pain or medium pain intensity” (VAS = 45–74), and “severe pain or high pain intensity” (VAS = 75–100) [22].

Pain-induced disability was assessed with the pain disability index (PDI). Global pain-induced disability is computed by the sum of pain-related disabilities in seven areas of daily life (family/home responsibilities, recreation, social activity, occupation, sexual behavior, self-care, and life-support activity), with each item ranging from 0 (no interference) to 10 (total interference). The global pain-induced disability index ranges from 0 to 70 [23]. In this study, participants were asked to answer the questionnaire regarding their current pain-induced disability (PDI_C_) and their pain disability from four weeks prior to the start of social distancing measures (PDI_P_).

Analgesic use, as either OTC or PO analgesic consumption, was assessed using a Likert scale. In order to assess the changes in analgesic use, the variables significantly increased use and increased use, as well as no changes, decreased use and significantly decreased use, were clustered.

The Patient Health Questionnaire for Depression and Anxiety (PHQ-4) was used to assess the psychological burden of the study group. Two items of the Patient Health Questionnaire for Depression (PHQ-2) and the Generalized Anxiety Disorder Scale (GAD-2) were combined to form PHQ-4 [24]. PHQ-4 is an overall screening tool for depression and anxiety. PHQ-2 and GAD-2 scores of ≥3 and ≥5 were considered as being discriminative and highly discriminative between the normal population and probable cases of major depression or generalized anxiety, respectively.

The Brief Resilience Scale (BRS) was used to assess how resilience affected the pattern of intake of analgesics. Smith et al. were the first to describe the BRS, which was developed to identify one’s ability to bounce back from stress [25].

### 2.2. Statistical Analysis

The study population was characterized by using descriptive statistics (frequencies, means, and standard deviations). Differences between study respondents and non-respondents were examined with χ^2^ tests and Mann–Whitney U tests. The statistical dependence between the rankings of two continuous variables expressed as a correlation coefficient ρ was assessed using Spearman correlations.

Univariate analyses were applied to find variables with proper discriminatory values for changes in OTC and PO analgesic use. The various demographic, pandemic-specific and disease-specific variables were used as possible independent predictors in a univariate analysis.

A multivariate regression analysis was performed with backward stepwise selection including the variables with *p*-values less than 0.25 in the univariate regression model [26,27]. Data were expressed as odds ratio (OR), variance (Nagelkerke R^2^), *p*-value, and 95% confidence interval (95% CI).

All of the tests were two-tailed, with a significance level of *p* < 0.05. All analyses were carried out with SPSS^®^ software Version 24 (IBM Corp. Released 2019. IBM SPSS Statistics for Windows, Version 24.0. Armonk, NY, USA).

## 3. Results

### 3.1. Demographic Characteristics

Our results were based on the data obtained from 278 subjects who answered at least one of the questions regarding changes in their consumption of analgesic self-medication (OTC vs. PO pain medication) during the first wave of the COVID-19 pandemic compared to the period before the pandemic. The sample represented 67.3% (278/413) of subjects who had accessed the web-based survey. The Supplementary Table S1 depicts the demographic characteristics of the study group (“Respondents”) compared to participants who did not answer any of the questions regarding the intake of pain medication. A Spearman analysis showed that increased intakes of either OTC pain medication or PO pain medication were correlated with one another (ρ = 0.595; *p* < 0.001).

### 3.2. Identification of Possible Predictors of Increased Intake of OTC and PO Analgesics in Women with Endometriosis

Univariate logistic regression analyses were used to assess the predictability of the selected independent variables on the odds of an increased intake of OTC and PO pain medication. Univariate analyses observing demographic predictors of the increased intake of OTC and PO analgesics did not show any significant association (all *p*-values > 0.05) (Supplementary Table S2).

**Figure 1:**
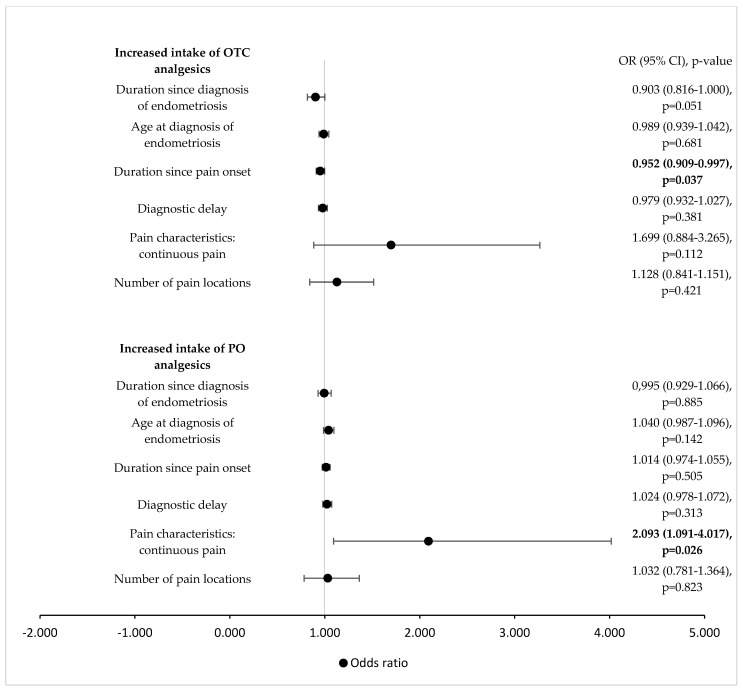
**Influence of endometriosis-specific factors on increased analgesic intake** (univariate logistic regression analysis). OTC = over the counter; PO = prescription only; OR = odds ratio; CI = confidence interval. Values in bold indicate statistical significance, as the level of statistical significance was set to *p* < 0.05.

**Figure 2:**
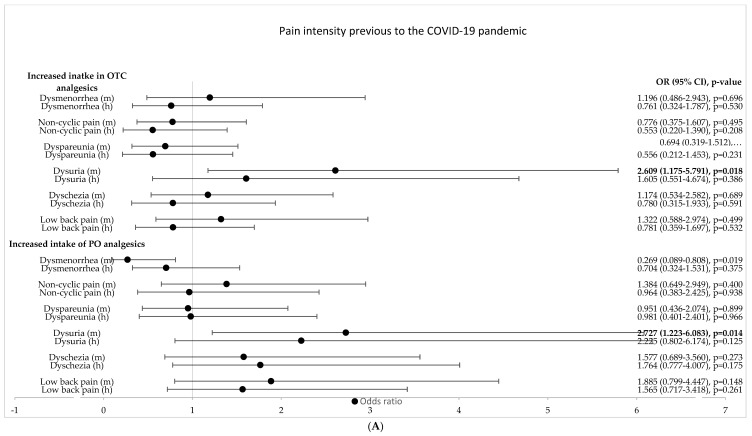

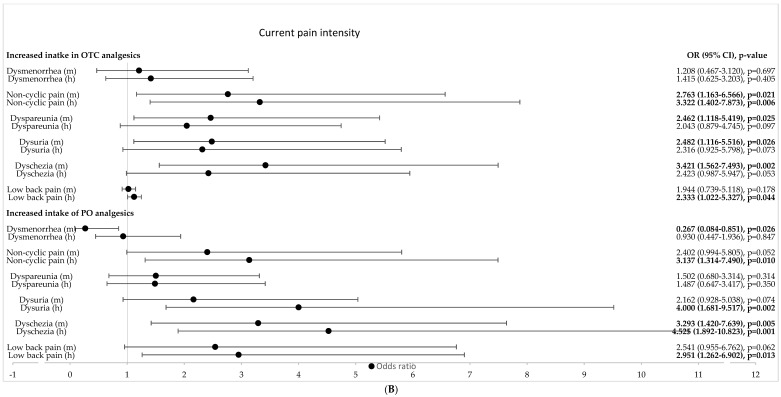
**Influence of pain level on increased intake of OTC and PO analgesics** (univariate logistic regression analysis). (**A**). Pain level previous to the COVID-19 pandemic. (**B**). Current pain level. OR = odds ratio; CI = confidence interval; m = medium pain intensity; h = high pain intensity. Values in bold indicate statistical significance; the level of statistical significance was set to *p* < 0.05.

**Figure 3:**
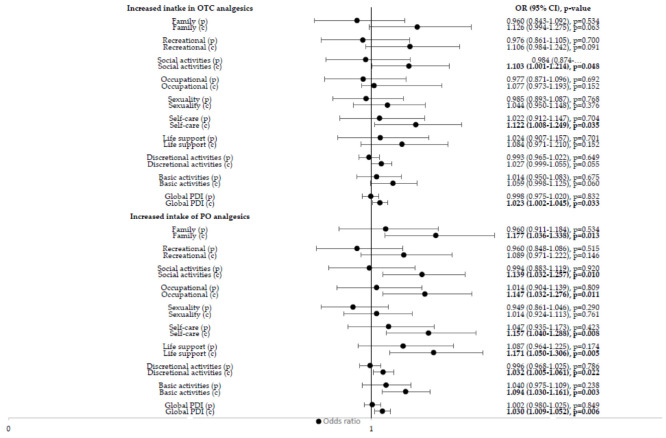
**Influence of pain-induced disability of daily life activities on increased intake of OTC and PO analgesics** (univariate logistic regression analysis). OTC = over the counter; PO = prescription only; OR = odds ratio; CI = confidence interval; p = previous to the COVID-19 pandemic; c = current. Values in bold indicate statistical significance, as the level of statistical significance was set to *p* < 0.05.

**Figure 4:**
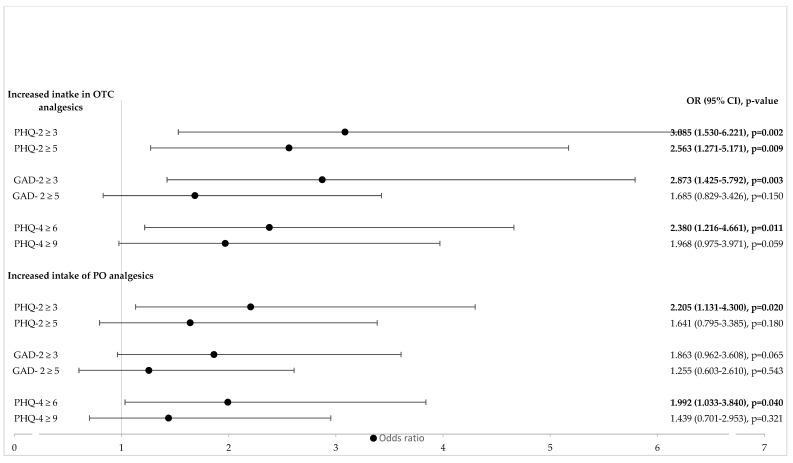
**Influence of mental health on increased intake of OTC and PO analgesics** (univariate logistic regression analysis). OTC = over the counter; PO = prescription only; OR = odds ratio; CI = confidence interval; GAD-2 = Generalized Anxiety Disorder Scale; PHQ-2 = Patient Health Questionnaire for Depression; PHQ-4 = Patient Health Questionnaire for Depression and Anxiety. Values in bold indicate statistical significance; the level of statistical significance was set to *p* < 0.05.

Among the investigated pandemic-specific factors, only the degree of reduction in social networks and the perceived reduction in social support were independent predictors of an increased intake of PO pain medication in the univariate analyses (Table 1). An increased duration since the onset of pain and experiencing continuous pain were associated with an increased intake of OTC and PO analgesics in the univariate analysis, respectively (Figure 1). With regard to pain intensity previous to the pandemic, only dysuria (medium pain intensity) increased the intake of OTC and PO (Figure 2A). A high level of current pain intensity (non-cyclic pain, dysuria, dyschezia and lower back pain) increased the odds of an increased intake of PO analgesics (Figure 2B). Current pain-induced disabilities with respect to activities of daily life, such as family activities, social activities, occupational activities, self-care and life support, as well as global pain-induced disability, were positively associated with an increased intake of PO analgesics in the univariate analysis (Figure 3. Pain-induced disability previous to the pandemic was not significantly associated with an increased intake of analgesics in the univariate analysis (Figure 3). Both anxiety and depression were positively associated with an increased intake of OTC analgesics, while only depression was associated with an increased intake of PO pain medication in the univariate analysis (Figure 4). Having a higher resilience was not associated with an increased intake of OTC analgesics (OR 1.080; 95% CI 0.726–1.605; *p* = 0.705) or PO pain medication (OR 0.934; 95% CI 0.628–1.387; *p* = 0.734).

### 3.3. Identification of Independent Predictors of Increased Intake of Analgesics by Women with Endometriosis

Multivariate logistic regression analyses, with the strongest predictors being the univariate analyses (*p* < 0.25) of increased intake of pain medication during the first wave of the COVID-19 pandemic, were utilized to assess the independence of the predicting variables (demographic, disease-specific, and pandemic-specific variables, as well as resilience). To avoid model overfitting and information redundancy, the current global pain-induced disability was included in the multivariate analysis, whereas the previous pain-induced disability or previous pain intensity were not involved. Current pain intensity was not considered in the following analyses because the correlation analyses revealed highly significant positive correlations between current global pain-induced disability and current pain intensity in this study population (Table 2).

The multivariate logistic regression model for the prediction of an increased intake of OTC analgesics included 260 women and considered the following possible predictors: age, educational level, duration since diagnosis of endometriosis, duration since pain onset, perceived reduction in social support during pain experience, continuous pain, current global pain-induced disability, PHQ-2 ≥ 3 and GAD-2 ≥ 3. Having a high educational level (OR 2.719; 95% CI 1.137–6.501; *p* = 0.025) and being at higher risk for depressive disorders as measured by PHQ-2 ≥ 3 (OR 2.398; 95% CI 1.055–5.450; *p* = 0.037) were found to be independent risk factors for an increased intake of OTC analgesics in women with endometriosis during the first wave of the COVID-19 pandemic. The proposed statistical model illustrated 13.6% of the variance. The model had a sensitivity of 84.4% for predicting an increased intake of OTC analgesics.

Educational level, reduction in social network, age at diagnosis of endometriosis, perceived reduction in social support during pain experience, continuous pain, current global pain-induced disability, PHQ-2 ≥ 3 and GAD-2 ≥ 3 were identified as possible predictors of an increased intake of PO analgesics in women with endometriosis. In the multivariate logistic regression model, only current global pain-induced disability (OR 1.030; 95% CI 1.007–1.054; *p* = 0.010) was identified as a risk factor for an increased intake of PO pain medication. The final regression model (*n* = 260) explained 10.9% of the variance and demonstrated a sensitivity of 83.5% for predicting an increased intake of PO analgesics.

## 4. Discussion

We showed previously that both OTC and PO analgesic use increased in 15.9% of study participants during the first wave of the pandemic, compared to pre-pandemic levels [5]. To our knowledge, this is the first study to investigate the predictive value of demographic, pandemic-specific and disease-specific factors, as well as mental health and resilience for an increased intake of analgesics in women with endometriosis. To date, most research concentrates on predictors of postoperative pain and/or opioid use in populations with various pain conditions other than endometriosis. Previous research has shown that the female sex and chronic diseases have been associated with self-medication in the general population [28,29]. In this study, age and relationship status did not influence OTC and PO analgesic use. Interestingly, having a higher education level was a risk factor for an increased intake of OTC pain medication in this study population. Fourquet and colleagues have already described the possible negative impact of endometriosis on work quality, including both presenteeism and absenteeism [30]. We hypothesize that women with a higher education level had a higher motivation to perform adequately at work, and as a result, they increased their OTC analgesic use. In line with our study, educational level has been shown to be a predictor of an increased use of OTC pain medication in a group of Spanish adults (aOR 1.89; 95% CI 1.37–2.62) [31]. Surprisingly, none of the pandemic-specific variables, such as the duration of reduction in social networks nor the degree of reduction in social networks, were independent risk factors for increased analgesic use in German women with endometriosis during the first wave of the COVID-19 pandemic in the multivariate logistic regression analyses.

Chronic pain is considered the leading cause of disability. Untreated chronic pain can cause job loss, economic problems, a depressive mood, and social isolation [32]. Pain intensity has been positively associated with an increased use of pain medications [16,29]. This study showed that being less able or unable to perform a range of daily activities, such as family-based, occupational and recreational activities, was the only independent risk factor for an increased intake of PO analgesics. Our results corroborate the previous findings by Kapadi et al., who reported a positive correlation between PO analgesic intake and pain severity [16]. In the current study, only increased pain-induced disability was independently associated with an increased intake of PO analgesics, while demographic, disease-specific, pandemic-specific factors, mental health and resilience were not. This might be related on one hand to a high level of responsibility regarding the use of PO pain medication in German women with endometriosis. On the other hand, as the survey was conducted during the first wave of the COVID-19 pandemic and medical facilities were inaccessible for patients without an emergency during that period, the use of PO analgesics might have been influenced and restricted by the government-imposed restraints on visiting a healthcare facility in order to collect an additional prescription, as well as by the anxiety of contracting the SARS-CoV-2 virus by attending a physician [32,33]. Similar factors associated with PO analgesic utilization have been reported previously. Functional disability, along with baseline pain and injury severity, was a predictive factor for long-term opioid use in a group of workers with lower back pain in the USA, while other demographic factors, such as age or education, were not [34]. Thus, measures to improve the ability to cope with pain and pain-induced disability might prevent an increased use of PO analgesics in women with endometriosis and mitigate the possible long-term adverse effects of the medication.

We showed that a depressive disorder was an independent risk factor for an increased intake of OTC analgesics. Psychological disorders (OR 2.24, 95% CI 1.81–2.78) have been recognized as risk factors of an increased intake of OTC analgesics in Spanish adults [31]. A systematic review has found a positive association between self-medication and depression in elderly persons [28]. Depression has been reported as a risk factor for OTC analgesic misuse in the general German population (OR = 1.73; 95% CI 1.34–2.24) [35]. As self-diagnosis is an acknowledged modifier and initiator of OTC intake [13], it might be possible that depressive symptoms influenced the perception of pain and pain-induced disability, and thus the need for pain relief. Pain and depression share common neurological pathways [36]. A large European survey revealed that 21% of study participants reported being diagnosed with depression as a result of a chronic pain disorder [11]. Depressive symptoms seem to play a major role in modifying the experiences and subsequent social and functional consequences of women with endometriosis [4]. Pain and depression might influence each other´s treatment, as has been shown in German patients with rheumatoid arthritis [37]. Living with pain during the stressful time of the pandemic could itself have been a traumatic experience. Moreover, the pandemic itself may have negatively influenced the physical and psychological well-being of individuals [38], and almost 50% of women with endometriosis showed scores in the PHQ-2 ≥ 3 [39]. Adequate psychological treatment could help to overcome catastrophic thinking over pain [40] and avoid the use of analgesics in order to treat emotional pain and affective distress.

Women with endometriosis often use analgesics as an appropriate adjustment to self-care in times when endocrine therapy is not appropriate or not recommended, e.g., in women wishing to get pregnant, or as an additional therapy to endocrine treatment and other recommended therapeutic options, such as acupressure, physical exercise, TENS units, etc. In order to identify women at risk of increased analgesic use and misuse, we recommend the assessment of pain-induced disability and mental health on a regular basis during treatment and follow-up. Women at risk for increased analgetic use could benefit from additional therapeutic options to relieve pain, such as yoga, physiotherapy and osteopathy [8]. Moreover, multidisciplinary treatment, including mental health specialists and clinical pharmacologists, should be considered in every woman with endometriosis in order to reduce pain, control short-term and long-term analgesic consumption and prevent the misuse of analgesics [8,40].

### Strengths and Limitations

The strengths of this study were the focus on women with endometriosis and the high number of participants who responded to the questions related to changes in their analgesic utilization. Moreover, we were able to assess a large number of potential influencing factors on analgesic consumption in women with endometriosis.

We also want to discuss the current findings in the context of some limitations. As the study participants were recruited by Facebook support groups of women with endometriosis, it might be possible that women suffering from more pain-induced disability or depression answered the questions regarding changes in their analgesic use. Nevertheless, according to a recent systematic review, Facebook-recruited samples are similarly representative to study samples recruited via traditional methods [41]. Additionally, we cannot identify the number of women who actually were aware of the study posted on the Facebook internet platform of the support groups of women with endometriosis, but decided not to access the questionnaire. Next, the lack of a control group and the design of the study (a non-randomized cross-sectional study) may have influenced representativeness. The results of this study described the risk factors of increased analgesic use in German women with endometriosis during the first wave of the COVID-19 pandemic, but these are not necessarily generalizable to women with endometriosis from other nationalities, nor to increased analgesic use during other life events or other time points during the COVID-19 pandemic.

Next, the instrument used to assess mental health, the PHQ-4 questionnaire, was more of a screening tool, rather than a diagnostic instrument, for core depression and anxiety disorder symptoms in the previous two weeks [24,42]. Nevertheless, PHQ-2 ≥ 3 has a sensitivity of 82.9% and a specificity of 90.0% for predicting a major depressive disorder [43], and GAD-2 ≥ 3 has a sensitivity of 86.0% and a specificity of 83.0% for predicting a generalized anxiety disorder [44]. We did not assess the specific analgesic medication of the study participants, but only the changes in pain medication. Additionally, we did not specifically assess whether the analgesics were used to attenuate pain only related to endometriosis. Moreover, recall bias and socially desirable responses may have influenced the results of the study. Nevertheless, as changes in life during the pandemic were consciously experienced by many, we conclude that these changes were more easily recalled than in other settings.

## 5. Conclusions

The COVID-19 pandemic changed the healthcare delivery for patients with chronic pain, as many appointments were cancelled or were provided by telemedicine. Women with endometriosis and depressive disorders or those reporting higher pain-induced disability were at a higher risk for an increased intake of analgesics. Understanding the underlying mechanisms of increased analgesic use when facing a stressful life event, such as the COVID-19 pandemic or other stressful life events, may help healthcare practitioners to decide who is at risk of increased analgesic use, misuse and dependency, and whether to see a patient in person and to address possible long-term adverse outcomes due to an analgesic use disorder. The ultra-brief PHQ-4 questionnaire, as well as the PDI questionnaire, could be widely implemented in both the inpatient and outpatient care of women with endometriosis in order to identify those at risk for increased analgesic intake. Longitudinal research is needed to better understand the individual factors associated with the risk for increased analgesic consumption in women with endometriosis.

## Figures and Tables

**Table 1:** Influence of pandemic-specific factors on changes in the intake of OTC and PO analgesics (univariate logistic regression analysis).

Increased Intake in OTC Analgesics		Increased Intake in PO Analgesics	
*p*-Value	OR (95% CI)	*p*-Value	OR (95% CI)
Duration of reduction in social network ≥15 days (co: <15 days)			
0.814	1.143 (0.376–3.472)	0.801	1.154 (0.380–3.506)
n.a.	n.a.	0.904	1.142 (0.134–9.722)
Large reduction in social network (co: not at all to moderate reduction in social network)			
0.490	1.308 (0.611–2.799)	**0.033**	**2.681** **(1.084–6.633)**
Perceived reduction in social support during pain experience (co: no reduction in social support)			
0.185	1.550 (0.810–2.966)	**0.010**	**2.375** **(1.234–4.571),**

OTC = over the counter; PO = prescription only; n.a. = not applicable; OR = odds ratio; CI = confidence interval; co = controls. Values in bold indicate statistical significance, as the level of statistical significance was set to *p* < 0.05.

**Table 2:** Correlation analysis (Spearman’s Rho) between PDI and pain characteristics.

Pain Characteristics		Dysmenorrhea (cv)	Non-Cyclic Pain (cv)	Dyspareunia (cv)	Dysuria(cv)	Dyschezia (cv)	Low Back Pain (cv)
**Global PDI**	Correlation coefficient	0.467	0.469	0.344	0.404	0.470	0.443
	*p*-value	<0.001	<0.001	<0.001	<0.001	<0.001	<0.001

PDI = pain-induced disability; cv = continuous variable.

## Data Availability

The data used to support the findings of this study are included within the article.

## Supplementary Materials

The following supporting information can be downloaded at: https://www.mdpi.com/article/10.3390/jcm11195520/s1, Table S1: Differences between participants who did not complete (group “Non-respondents”) vs. those who completed the questions regarding analgesic intake (group “Respondents”); Table S2: Influence of demographic factors on the intake of OTC and PO analgesics (univariate logistic regression analysis).
