# Supplementary material for: Factors Associated with Increased Analgesic Use in German Women with Endometriosis during the COVID-19 Pandemic
Source: J Clin Med. 2022 Sep 21;11(19):5520. doi: 10.3390/jcm11195520 (PMC9570957; doi:10.3390/jcm11195520)
Supplement: Supplementary file 1 [file jcm-11-05520-s001.zip › jcm-1922526-supplementary.pdf]

**Table S1. Differences between participants who did not complete (group "Non-respondents") versus those who completed the questions regarding analgesic intake (group "Respondents").**

| Variables | Values | Non-respondents | Respondents | p-value |
|---|---|---|---|---|
| **Demographic variables** | | | | |
| **Age** | M (SD); N<br>Mdn (IQR) | 32.36 (6.85); 115<br>32.00 (27.00 – 37.00) | 31.97 (7.13); 278<br>31.00 (26.00 – 36.00) | 0.588[2] |
| **Having a stable relationship** | No in % (n/N)<br>Yes in % (n/N) | 72.3% (73/101)<br>27.7% (28/101) | 77.7% (216/278)<br>22.3% (62/278) | 0.273[1] |
| **Living alone** | No in % (n/N)<br>Yes in % (n/N) | 74.1% (86/116)<br>25.9% (30/116) | 79.8% (221/277)<br>20.2% (56/276) | 0.217[1] |
| **Educational level** | Up to secondary level in % (n/N)<br>Tertiary level in % (n/N) | 50% (1/2)<br>50% (1/2) | 29.2% (78/267)<br>70.5% (189/267) | 0.502[3] |
| **Pandemic-specific variables** | | | | |
| **Duration of i/q** | M (SD); N<br>Mdn (IQR) | 24.80 (13.48)<br>21.00 (20.00-30.00) | 27.76 (11.92)<br>27.00 (21.00-32.00) | 0.004[2] |
| **Duration of i/q** | <15d in % (n/N)<br>≥15d in % (n/N) | 17.0% (17/100)<br>83.0% (83/100) | 10.1% (28/278)<br>89.9% (250/278) | 0.067[1] |
| **Being in i/q** | No in % (n/N)<br>Yes in % (n/N) | 6.1% (7/115)<br>93.9% (108/115) | 2.5% (7/278)<br>97.5% (271/278) | 0.082[1] |
| **Reduction in social network** | No to moderate reduction in % (n/N)<br>Large reduction in % (n/N) | 32.7% (33/101)<br>67.3% (68/101) | 27.0% (75/278)<br>73.0% (203/278) | 0.278[1] |
| **Perceived reduction in social support regarding pain experience during social isolation (by partner/ family/ friends)** | No in % (n/N)<br>Yes in % (n/N) | 100.0% (1/1)<br>0.0% (0/1) | 61.2% (169/276)<br>38.8% (107/276) | 0.427[1] |
| **Endometriosis-specific variables** | | | | |
| **Time since diagnosis (y)** | M (SD); N<br>Mdn (IQR) | 4.07 (4.76); 94<br>2.00 (1.00 – 5.00) | 4.37 (4.79); 277<br>3.00 (1.00 – 5.00) | 0.360[2] |
| **Age at diagnosis (y)** | M (SD); N<br>Mdn (IQR) | 28.26 (6.71); 94<br>28.50 (24.00 – 33.00) | 27.62 (6.25); 277<br>27.00 (23.00 – 32.50) | 0.268[2] |
| **Time since pain onset (y)** | M (SD); N<br>Mdn (IQR) | 13.28 (7.59); 96<br>12.00 (7.00 – 18.50) | 14.00 (7.88); 278<br>13.00 (8.00 – 20.00) | 0.470[2] |
| **Diagnostic delay (y)** | M (SD); N<br>Mdn (IQR) | 9.31 (6.86); 94<br>9.00 (5.00 – 14.00) | 9.65 (6.91); 277<br>9.00 (4.50 – 14.00) | 0.844[2] |
| **Pain characteristics** | Pain peaks in % (n/N)<br>Continuous pain in % (n/N) | 64.2% (61/95)<br>35.8% (34/95) | 65.1% (181/278)<br>34.9% (97/278) | 0.874[1] |

| | | | | |
|---|---|---|---|---|
| **Number of pain localizations** | M (SD); N<br>Mdn (IQR) | 5.18 (0.95); 38<br>5.00 (5.00 – 6.00) | 5.05 (1.15); 277<br>5.00 (5.00 – 6.00) | 0.784[2] |
| **Pain intensity** | | | | |
| **Dysmenorrhea prior to i/q** | M (SD); N<br>Mdn (IQR) | 63.58 (32.35); 36<br>72.50 (45.00 – 91.50) | 65.25 (31.21); 248<br>73.50 (46.50 – 90.00) | 0.813[2] |
| **Non-cyclical pain prior to i/q** | M (SD); N<br>Median (IQR) | 45.58 (31.02); 38<br>41.00 (20.00 – 71.00) | 51.89 (26.64);265<br>51.00 (32.00 – 72.00) | 0.163[2] |
| **Dyspareunia prior to i/q** | M (SD); N<br>Mdn (IQR) | 39.56 (33.06); 32<br>30.50 (10.00 – 64.50) | 45.07 (32.53); 252<br>45.50 (14.50 – 69.00) | 0.391[2] |
| **Dysuria prior i/q** | M (SD); N<br>Mdn (IQR) | 26.94 (32.56); 32<br>9.50 (3.00 – 47.50) | 28.97 (28.95); 247<br>20.00 (3.00 – 48.00) | 0.488[2] |
| **Dyschezia prior to i/q** | M (SD); N<br>Mdn (IQR) | 36.35 (30.31); 34<br>26.50 (10.00 – 58.00) | 41.07 (31.17); 259<br>37.00 (13.00 – 68.00) | 0.414[2] |
| **Lower back pain prior to i/q** | M (SD); N<br>Mdn (IQR) | 54.84 (32.38); 37<br>48.00 (33.00 – 89.00) | 57.69 (32.42); 270<br>60.00 (33.00 – 88.00) | 0.529[2] |
| **Current dysmenorrhea** | M (SD); N<br>Mdn (IQR) | 32.75 (41.36); 4<br>22.50 (0.00 – 65.50) | 60.25 (33.63); 253<br>70.00 ( 30.00 – 87.00) | 0.118[2] |
| **Current non-cyclical pain** | M (SD); N<br>Mdn (IQR) | 38.33 (42.11); 6<br>21.00 (5.00 – 82.00) | 52.79 (30.02); 268<br>56.00 (28.50 – 77.50) | 0.295[2] |
| **Current dyspareunia** | M (SD); N<br>Mdn (IQR) | 44.50 (43.61); 4<br>46.50 (7.00 – 82.00) | 44.03 (35.41); 253<br>45.00 (8.00 – 73.00) | 0.938[2] |
| **Current dysuria** | M (SD); N<br>Mdn (IQR) | 4.67 (35.63); 3<br>3.00 (1.00 – 10.00) | 29.47 (31.04); 253<br>16.00 (2.00 – 51.00) | 0.249[2] |
| **Current dyschezia** | M (SD); N<br>Mdn (IQR) | 21.67 (35.81); 3<br>2.00 (0.00 – 63.00) | 40.37 (32.33); 258<br>37.00 (10.00 – 67.00) | 0.217[2] |
| **Current lower back pain** | M (SD); N<br>Mdn (IQR) | 34.40 (23.46); 5<br>24.00 (20.00 – 34.00) | 58.85 (34.11); 266<br>64.50 (29.00 – 89.00) | 0.110[2] |
| **Pain-induced disability** | | | | |
| **Family prior to i/q** | M (SD); N<br>Mdn (IQR) | 5.78 (2.33); 36<br>6.00 (5.00 – 8.00) | 5.12 (2.48); 278<br>5.00 (3.00 – 7.00) | 0.154[2] |
| **Recreational prior to i/q** | M (SD); N<br>Mdn (IQR) | 5.75 (2.52); 36<br>6.00 (4.00 – 8.00) | 5.57 (2.57); 278<br>6.00 (4.00 – 8.00) | 0.705[2] |
| **Social activities prior to i/q** | M (SD); N<br>Mdn (IQR) | 5.86 (2.90); 36<br>6.00 (3.00 – 8.00) | 5.42 (2.72); 278<br>6.00 (3.00 – 8.00) | 0.324[2] |
| **Occupational prior to i/q** | M (SD); N<br>Mdn (IQR) | 6.50 (2.68); 36<br>7.00 (4.00 – 8.50) | 5.97 (2.80); 278<br>6.00 (4.00 – 8.00) | 0.306[2] |
| **Sexuality prior to i/q** | M (SD); N<br>Mdn (IQR) | 6.20 (3.27); 35<br>7.00 (3.00 – 9.00) | 6.00 (3.26); 278<br>7.00 (3.00 – 9.00) | 0.721[2] |
| **Self-care prior to i/q** | M (SD); N | 2.72 (3.16); 36 | 2.74 (2.77); 278 | 0.698[2] |

| | | | | |
|---|---|---|---|---|
| | Mdn (IQR) | 1.00 (0.00 – 5.00) | 2.00 (0.00 – 5.00) | |
| **Life support prior to i/q** | M (SD); N<br>Mdn (IQR) | 2.64 (2.95); 36<br>2.00 (0.00 – 5.00) | 2.68 (2.61); 278<br>2.00 (0.00 – 5.00) | 0.583[2] |
| **Discretional activities prior to i/q** | M (SD); N<br>Mdn (IQR) | 30.00 (10.90); 35<br>31.00 (23.00 – 38.00) | 28.04 (11.14); 274<br>29.50 (20.00 – 37.00) | 0.355[2] |
| **Basic activities prior to i/q** | M (SD)<br>Mdn (IQR) | 5.37 (5.59); 35<br>4.00 (0.00 – 9.00) | 5.42 (4.86); 274<br>4.00 (1.00 – 9.00) | 0.618[2] |
| **Global PDI prior to i/q** | M (SD); N<br>Mdn (IQR) | 35.37 (15.12); 35<br>36.00 (24.00 – 46.00) | 33.46 (14.26); 278<br>34.00 (23.00 – 43.00) | 0.582[2] |
| **Current family activities** | M (SD); N<br>Mdn (IQR) | 3.00 (1.73); 5<br>2.00 (2.00 – 3.00) | 5.35 (2.69); 277<br>5.00 (3.00 – 8.00) | **0.041**[2] |
| **Current recreational activities** | M (SD); N<br>Mdn (IQR) | 2.40 (2.07); 5<br>2.00 (1.00 – 4.00) | 5.37 (2.89); 277<br>6.00 (3.00 – 8.00) | **0.024**[2] |
| **Current social activities** | M (SD); N<br>Mdn (IQR) | 3.00 (3.94); 5<br>1.00 (0.00 – 5.00) | 4.52 (3.43); 276<br>5.00 (1.00 – 8.00) | 0.324[2] |
| **Current occupational activities** | M (SD); N<br>Mdn (IQR) | 1.80 (2.05); 5<br>1.00 (0.00 – 4.00) | 5.34 (3.25); 276<br>5.00 (3.00 – 8.00) | 0.019[2] |
| **Current sexuality** | M (SD); N<br>Mdn (IQR) | 5.50 (4.80); 4<br>6.00 (1.50 – 9.50) | 5.52 (3.48); 274<br>6.00 (3.00 – 9.00) | 0.960[2] |
| **Current self-care** | M (SD); N<br>Mdn (IQR) | 1.00 (1.41); 5<br>0.00 (0.00 – 1.00) | 2.86 (2.85); 277<br>2.00 (0.00 – 5.00) | 0.130[2] |
| **Current life support** | M (SD); N<br>Mdn (IQR) | 0.80 (1.30); 5<br>1.00 (0.00 – 1.00) | 2.70 (2.78); 277<br>2.00 (0.00 – 5.00) | 0.113[2] |
| **Current discretional activities** | M (SD); N<br>Mdn (IQR) | 17.00 (12.73); 4<br>18.50 (6.50 – 27.50) | 26.18 (12.51); 273<br>27.00 (16.00 – 36.00) | 0.163[2] |
| **Current basic activities** | M (SD); N<br>Mdn (IQR) | 2.00 (2.83); 4<br>1.00 (0.00 – 4.00) | 5.61 (5.12); 273<br>5.00 (1.00 – 9.00) | 0.130[2] |
| **Current global PDI** | M (SD); N<br>Mdn (IQR) | 19.00 (14.90); 4<br>21.00 (6.50 – 31.50) | 31.79 (15.79); 273<br>32.00 (19.00 – 43.00) | 0.124[2] |
| **Mental outcomes** | | | | |
| **PHQ-2** | M (SD); N<br>Mdn (IQR) | 0.00 (0.00); 2<br>0.00 (0.00 – 0.00) | 2.85 (1.68); 272<br>2.00 (2.00 – 4.00) | **0.017**[2] |
| **GAD-2** | M (SD); N<br>Mdn (IQR) | 0.50 (0.71); 2<br>0.50 (0.00 – 1.00) | 2.90 (1.82); 272<br>2.00 (2.00 – 4.00) | **0.044**[2] |
| **PHQ-4** | M (SD); N<br>Mdn (IQR) | 0.50 (0.71); 2<br>0.50 (0.00 – 1.00) | 5.75 (3.19); 272<br>5.00 (3.00 – 8.00) | **0.020**[2] |
| **Resilience (BRS)** | M (SD); N<br>Mdn (IQR) | 2.66 (1.45); 3<br>3.33 (1.00 – 3.66) | 2.75 (0.82); 270<br>2.66 (2.16 – 3.33) | 0.837[2] |

BRS= brief resilience score; GAD-2= Generalized Anxiety Disorder Scale; PHQ-2= Patient Health Questionnaire for Depression; PHQ-4= Patient Health Questionnaire for Depression and Anxiety; i/q=isolation or quarantine; d= days; N= Number of women for which data were available; n= sample size; M=mean; SD= standard deviation, Mdn= median; IQR: Interquartile Range; n.a.= not available/not applicable; y=years, Values in bold indicate statistical significance, as the level of statistical significance was set to $p<0.05$ ([1]=$\chi^2$ -test; [2]=Mann-Whitney-U-test; [3]=Fisher exact test).

**Table S2. Influence of demographic factors on the intake of OTC and PO analgesics (univariate logistic regression analysis).**

| Increased intake in OTC pain medication | | Increased intake in PO pain medication | |
|---|---|---|---|
| p-value | OR (95% CI) | p-value | OR (95% CI) |
| **Age** (in years) | | | |
| 0.078 | 0.958 (0.913–1.005) | 0.270 | 1.025 (0.981–1.072) |
| **Having a partner** (co: not having a partner) | | | |
| 0.475 | 0.741 (0.325–1.689) | 0.534 | 0.769 (0.337–1.757) |
| **Living alone** (co: not living alone) | | | |
| 0.775 | 0.886 (0.386–2.033) | 0.508 | 0.745 (0.312–1.779) |
| **Tertiary educational level** (co: up to secondary educational level) | | | |
| 0.119 | 1.919 (0.845–4.359) | 0.061 | 0.526 (0.269–1.029) |

OTC = 0ver-the-counter; PO = prescription only; OR = odds ratio; CI = confidence interval; co = controls.
